# The Needs of School Professionals for Eating Disorder Prevention in Australian Schools: A Mixed-Methods Survey

**DOI:** 10.3390/children9121979

**Published:** 2022-12-16

**Authors:** Kirrilly M. Pursey, Melissa Hart, Alexis Hure, Hei Man Cheung, Liting Ong, Tracy L. Burrows, Zali Yager

**Affiliations:** 1 School of Health Sciences, College of Health, Medicine and Wellbeing, University of Newcastle, Callaghan 2308, Australia; 2 Food and Nutrition Research Group, Hunter Medical Research Institute, New Lambton Heights 2305, Australia; 3 Hunter New England Mental Health, Mental Health Administration Building, Waratah 2298, Australia; 4 School of Medicine and Public Health, College of Health, Medicine and Wellbeing, University of Newcastle, Callaghan 2308, Australia; 5 Health Research Economic, Hunter Medical Research Institute, New Lambton Heights 2305, Australia; 6 Body Confident Collective, Melbourne 3011, Australia; 7 Institute for Health and Sport, Footscray Park Campus, Victoria University, Melbourne 3011, Australia

**Keywords:** eating disorders, body image, prevention, schools, teachers

## Abstract

(1) Background: School professionals such as teachers and counsellors are uniquely positioned to facilitate discussion around disordered eating and body image; however, little is known about the needs of school professionals with respect to eating disorder prevention. This study aimed to explore the needs and perceptions of Australian school professionals regarding eating disorder prevention. (2) Methods: School professionals were recruited to a mixed-methods online cross-sectional survey. The survey assessed demographics and perceived needs and attitudes to eating disorder prevention. (3) Results: Most participants (92%) were willing to participate in eating disorder prevention; however, only 61% reported good knowledge and 41% reported feeling confident in implementing eating disorder prevention. Those who had received training in eating disorders (24%) reported higher confidence (*p* = 0.02) and knowledge (*p* = 0.04). Only 66% of respondents reported that all teachers should be involved in eating disorder prevention while barriers including workload, knowledge, and resources were commonly highlighted. Fewer respondents working in primary school settings reported the need for prevention approaches (*p* = 0.046). (4) Conclusions: Despite a willingness to be involved in the prevention of eating disorders, there are inconsistencies in attitudes regarding the role of school professionals in eating disorder prevention. The findings of this study reinforce that understanding professional roles, school settings, and personal attitudes is critical in the development of more efficacious school professional training and prevention interventions.

## 1. Introduction

Eating disorders are complex mental illnesses that can result in long-term medical complications and premature death. Globally, the lifetime prevalence of eating disorders is 8.4% [1], while in Australia, the point prevalence of any eating disorder in adolescents is estimated to be 22% [2]. Concerningly, body image concerns, depression, anxiety, and eating disorder symptoms have increased by more than 50% during the COVID-19 pandemic [3,4]. Eating disorders can develop at any age; however, evidence has shown that young people, including children and adolescents, are at a higher risk [5,6,7]. Body image concerns and eating disorders are particularly concerning in children and adolescents as they can have bidirectional effects with cognitive, social and psychological aspects of health [8]. In addition, the presence of disordered eating at the preadolescent stage confers a strong risk of eating disorder in adolescence and early adulthood [9,10,11,12]. Therefore, early recognition and evidence-based intervention for eating disorders are essential.

Body dissatisfaction and weight-control behaviours, such as dieting, are among the strongest contributing risk factors for developing eating disorders [13,14,15,16]. Approximately half of children aged 6 to 12 years are dissatisfied with their body [17,18], while weight concerns and issues around ideal body types have been reported from the age of five [17,19,20]. This suggests that children of school age (5–18 years) are in a critical window of need for prevention and early intervention approaches, and schools are recognised as important settings for this work. However, despite 20 years of body image and eating disorder prevention programs being implemented and evaluated, while systematic reviews have revealed small positive effects in body image, no programs have demonstrated consistent efficacy for both boys and girls [21,22]. Interestingly, both reviews reported that programs were predominantly implemented by external providers as opposed to school professionals, and yet teacher and peer delivery of programs is recognised as being more effective [23,24]. Health and Physical Education curricula allows for the delivery of education that can support the development of positive associations with food and bodies, and yet many students report that the education they received was either not sufficient or was more problematic than helpful [25,26].

School professionals such as teachers, counsellors, and psychologists play an important role in providing support to students. School professionals have a unique opportunity for interactions with students, which places them in an advantageous position to facilitate appropriate discussion regarding nutrition, exercise and body image as well as early identification of problematic attitudes and behaviours [25]. Eating disorders and their associated risk factors, such as dieting and body image, are often a sensitive topic to discuss with students. School professionals may therefore require guidance on how to approach these subjects and how to work positively with the student and their parents. A study conducted in the UK found that school professionals felt ill equipped to identify and support students with eating disorders [27]. Moreover, there have been some studies that indicate knowledge and resources might be a barrier to effective prevention in the school setting [27,28]. The success and sustainability of health-promotion programs in schools have been found to be highly dependent on teachers’ commitment and confidence to health education. Belief in their own professional effectiveness, positive attitude towards their role in promoting health, and perceptions of health promotion programs’ effectiveness and acceptability can create a favourable environment [29]. Hence, understanding school professionals’ knowledge and attitudes towards eating disorder prevention is important to inform future prevention program development, uptake, and effectiveness [30].

Given the critical need for prevention education and potential positive impact of teachers and school professionals, investigation into the knowledge and skill gaps that prevent effective action and intervention in terms of body image and eating disorders is warranted. Currently, little is known about the needs and attitudes of school professionals with respect to eating disorder prevention, particularly within an Australian context. School professionals’ self-perceived knowledge of eating disorders, as well as confidence and willingness to implement eating disorder prevention, is also currently under-represented in the literature. This is important to better understand perceived training and education needs as well as potential barriers to the implementation of prevention interventions within the school context. In view of the potential for regional differences in resources, it is also important to consider regional specificity when assessing the needs of teachers to ensure the effective development and implementation of programs for a specific context. Finally, most research investigating the needs of teachers to date has focused on the management and support of students experiencing eating disorders [27], with a paucity of studies investigating prevention of eating disorders, indicating a need for further evidence in this area.

Identifying the needs and perceptions of school professionals with respect to eating disorder prevention is a critical step towards developing efficacious and sustainable prevention interventions that can be implemented in the school setting, particularly in regional contexts. Therefore, the current pilot study aimed to evaluate the needs and perceptions of Australian school professionals in regional New South Wales regarding the implementation of eating disorder prevention approaches.

## 2. Materials and Methods

### 2.1. Study Design

This was a mixed-methods online cross-sectional survey designed to assess the needs and perceptions of school professionals with respect to the prevention of eating disorders, focusing on body image concerns and extreme weight control behaviours. The survey was approved by the University of Newcastle Human Ethics Committee (Approval number H-2017-0377; approval date 9 February 2018). This study was conducted in accordance with the Declaration of Helsinki.

### 2.2. Participants

Professionals currently working in schools and over 18 years of age were eligible to participate in the study. Eligible school professionals could include, but were not limited to, classroom teachers, health and physical education teachers, head teachers, welfare support, special needs teachers, school counsellors, and assistant principals or principals. Exclusion criteria included individuals not currently working in schools and those aged <18 years of age. The participant information statement was presented on the first page, with a question asking if the individual would like to participate in the survey at the bottom of the page. By clicking “Yes” to enter the survey, consent to participate was provided. All participants were screened to meet the inclusion and exclusion criteria with a question at the start of the survey. The survey was anonymous, unless participants chose to enter their contact details for participation in future research at the end of the survey.

### 2.3. Measures

The survey questions were developed by the research team for the purpose of this needs assessment. Attitude and perception questions were based on a previous report conducted in Victoria, Australia, in 2010 which assessed attitudes towards professional development and preparedness for body image education [31]. School representatives across a regional geographical area (*n* = 6) were consulted for feedback regarding the draft questions. The survey was then pilot tested for readability and clarity with a convenience sample of five clinicians and researchers working in the area of eating disorders and body image. Minor adjustments were made by the research team to address issues related to the terminology, flow of questions, and clarity of instructions.

The final 31-item survey took 15–20 min to complete and was comprised of demographic questions and items to determine school professionals’ perceptions and needs with respect to eating disorder prevention. Demographic questions with fixed responses included participant sex, age, years of experience in the school setting, the type of school they were working at, and current role in the school. A range of direct questions were asked regarding the perceived confidence, competence, and willingness to implement prevention approaches, the perceived priority of eating disorder prevention at their school, barriers to eating disorder prevention, and attitudes towards eating disorder prevention. Participants responded to these items on a 5-point Likert scale (*Strongly Agree* to *Strongly Disagree*). Perceived-needs questions included perceived requirement for prevention approaches in their school, preferred mode of delivery of prevention interventions, and training needs for school professionals. Question response formats for perceived needs included a combination of multiple-choice, open-ended, or five-point Likert-scale ratings. Open-ended responses were used to explore perceptions and needs in richer detail. All questions required a response for the survey to progress, minimising missing data, and followed a logic sequence to direct participants to certain questions based on their response. Study data were collected and managed using REDCap electronic data capture tools hosted by the Hunter Medical Research Institute [32,33]. REDCap (Research Electronic Data Capture) is a secure, web-based software platform designed to support data capture for research studies.

### 2.4. Procedure

Participants were recruited via social media posts advertised on the University of Newcastle’s Physical Activity and Nutrition Priority Research Centre social media page. “Virtual snowballing” was used, whereby the survey link was shared by participants with other potentially interested participants. In addition, emails containing the survey link were disseminated via the Hunter New England Mental Health SchoolLink coordinators. The survey link was open for a three-month period for completion from July to September in 2020.

### 2.5. Data Analysis

Surveys with no questions completed (*n* = 7) and those with only demographic information completed (*n* = 9) were excluded from the analysis. The data were exported from REDCap for analysis in Stata13 [34]. Data were checked for normality and reported as medians due to a skewed distribution. Categorical data were analysed descriptively and reported as frequencies and percentages. Due to low cell counts, ‘*Disagree*’ and ‘*Strongly disagree*’ responses were grouped, and ‘*Agree*’ and ‘*Strongly agree’* responses were grouped for analysis. Participants were grouped by school setting (primary or secondary school) for sub-analysis. Fisher’s exact test was used to assess associations between professionals’ perceptions (e.g., self-perceived knowledge and confidence) according to the school setting and whether the participant had received any training in eating disorders. Open-ended responses were analysed using NVivo. An inductive approach was taken to allow key elements to arise from the data. These elements were used to generate initial codes, which were then grouped into themes by two researchers and were reviewed by the research team. Quantitative and qualitative data were triangulated to identify similar themes. Qualitative data were grouped according to according to school setting (i.e., primary or secondary school) for synthesis and comparison. Participant identifiers were removed and replaced with a numerical code ranging from R1 to R67.

## 3. Results

### 3.1. Participant Characteristics

A total of 51 participants completed the survey. There were no significant differences in participant characteristics between survey completers (*n* = 51) and non-completers (i.e., those who only completed demographic questions, *n* = 9). Participants were predominantly female (86%) with a median age of 41 years (Table 1). Forty percent of participants reported post-graduate qualifications with years of teaching experience ranging from 0.5 to 38 years (median = 9 years). Forty-nine percent of respondents were working in a secondary school only, followed by working across both primary and secondary (29%), and primary schools only (22%). Most participants were working in government schools (78%) in a co-educational setting (98%). Participants held a range of school roles which included, in descending order, classroom teacher (43%), counsellor or psychologist (20%), welfare or wellbeing team (18%), or executive (Head Teacher (10%), Assistant or Deputy Principal (6%), and Principal (4%)). Only 24% of respondents reported that they had received training regarding eating disorders, which included education from an external provider, education during their degree, or self-directed learning. When analysed by school setting (i.e., primary or secondary school), fewer primary school professionals had received eating-disorder-related training compared to secondary school professionals (*p* = 0.04).

### 3.2. School Professionals’ Perceptions and Attitudes towards Eating Disorder Prevention

More than half (*n* = 31; 61%) of respondents rated their knowledge of eating disorders as *Good* or *Excellent*, and nearly all (*n* = 47; 92%) were *Willing* or *Extremely willing* to participate in eating disorder prevention (Table 2). Most respondents (*n* = 41; 80%) perceived eating disorder prevention to be part of their role at school; however, less than half (*n* = 21; 41%) reported feeling confident in implementing prevention approaches. When analysed according to whether the participant had received eating disorder training, a greater proportion of those who had received training reported *Good* to *Excellent* knowledge (*p* = 0.04) and confidence (*p* = 0.02). There was no difference in the willingness to participate in eating disorder prevention approaches according to whether training had been received.

Most respondents (*n* = 47; 92%) reported that eating disorder prevention programs were needed in schools, but only 12 respondents (25%) reported that their schools had current programs in place (Table 2). Such interventions outlined in the open-ended responses included sessions delivered by external providers (e.g., The Butterfly Foundation) and health and wellbeing programs specifically targeted at girls. When analysed by school setting (i.e., primary or secondary school), fewer respondents working in primary school settings reported the need for prevention approaches in their school compared to secondary schools (*p* = 0.046), and fewer primary schools had current prevention approaches in place (*p* = 0.03).

Eating disorder prevention was not perceived by participants as a high priority in the school in which they worked (median 4 out of a possible 10), and only 12 (25%) reported appropriate structures or policies to support eating disorder prevention in schools. In the open-ended responses elaborating on existing structures and policies in place at schools, participants specified wellbeing programs, personal development health and physical education (PDHPE) curricula, and policies to support mental health risks as appropriate structures so support the implementation of eating disorder prevention programs. “…PDH programs and welfare structures are solid for supporting individuals but this not addressed explicitly to whole grades/stages.”—R8 (Primary). “We have a referral system in place for students that we think are at risk and then work from a perspective of whole class or small group instruction.”—R39 (Primary and Secondary).

A total of 12 respondents (24%) perceived that there could be a potential negative impact related to the implementation of eating disorder prevention at schools, while a further 21 (41%) were unsure whether there would be negative consequences. Open-ended responses expanding on the perceived consequences revealed that school professionals perceived that there could be an increased awareness of body image and disordered eating behaviours, which may in turn foster the development of these behaviours and attitudes. Interestingly, these themes around consequences were more commonly reported my those in primary school settings. “May draw attention to what extreme dieting can result in- having an opposite effect.”—R30 (Primary). However, it was acknowledged in some of the responses that the benefits of prevention outweighed the potential negative consequences. “Increased awareness of students that peers are struggling with their body image and this could raise questions that some students had not considered previously. Yet, I do see the benefits outweighing this.” R8 (Primary)

School professionals generally had a positive attitude towards eating disorder prevention and their professional role in this. Most respondents (*n* = 43; 88%) disagreed that eating disorder prevention should be aimed at vulnerable or at-risk students only but agreed that it should be a whole-of-school approach (*n* = 38; 81%). Two-thirds (*n* = 32; 66%) of the respondents agreed that all teachers should be involved in eating disorder prevention and *n* = 43 (88%) believed teachers should act as role models for healthy eating behaviours and positive body image in school. However, some barriers to eating disorder prevention were identified by participants. From the predefined response list, the primary barriers to implementing eating disorder prevention, as indicated by *Agreement* or *Strong Agreement*, were: workload (75%), knowledge (75%), lack of appropriate resources (73%), and funding (52%). Participants from primary school settings were more likely to strongly agree with barriers to the implementation of eating disorder prevention approaches such as workload (*p* = 0.049), resources (*p* = 0.01), and funding (*p* = 0.04) compared to secondary school professionals. Although barriers were not assessed specifically as part of the open-ended questions, barriers including workload, knowledge, and resources were also highlighted in the final open-ended question about whether the participant had anything further to add, reinforcing the importance of considering these when developing prevention programs. “The content I need to get through is A LOT. … If I am to deliver education around eating disorders, I want the information supplied as it’s not my area and I would hate to say/do the wrong thing.”—R13 (Secondary). “Really lack of time due to case load… Our job is very reactive due to high demand of students and staff.”—R5 (Primary and Secondary).

### 3.3. Needs Regarding the Implementation of Eating Disorder Prevention and School Professionals Training

Participants reported that physical activity (88%) and healthy eating (81%) were commonly taught in schools, predominantly through the PDHPE curriculum. However, topics such as body image (46%), unhealthy weight control behaviours (27%) and media literacy (27%) were reported as less commonly taught, even though these were acknowledged as important areas. In addition, modes of delivery and target groups for these topics appeared more varied throughout the open-ended responses. “These concepts are embedded in PDHPE lessons but personally I don’t think we spend enough time on them. Unfortunately, the time we have to cover pertinent issues is limited.” R55 (Secondary). However, a common theme that emerged across many respondents, particularly among secondary school respondents, included identifying opportunities to embed this information as part of existing structures such as PDHPE, welfare and wellbeing programs. “We have established wellbeing programs within the school that are coordinated by our wellbeing team and could easily integrate a program focused on body image”—R6 (Secondary).

When school professionals were asked about what they perceived would be the most effective mode of delivery for eating disorder prevention in their school, a range of responses were expressed including the provision of training and resources to teachers, incorporation of materials into the curriculum, and delivery by external providers. Overwhelmingly, though, an emerging theme was the multimodal delivery of these aspects. “I believe teacher education for them to be able to incorporate it as part of curriculum is necessary. Information sessions/days from external providers would be good addition.”—R5 (Primary and Secondary). “Providing training and resources to teachers. I also see a benefit in external providers educating students… They would benefit from someone who they could ‘relate’ to.”—R8 (Primary). Although teacher training was highlighted as a key area for delivery, in the open-ended responses, the need for appropriate teacher training in this area was highlighted. “Getting teachers with minimal training or a token amount of training to deliver programs to these at-risk students could actually be counterproductive.”—R55 (Secondary).

In the open-ended responses assessing training needs, support for professional training in eating disorder prevention was highly endorsed by participants. Regarding the preferred mode of delivery, face-to-face workshops and online delivery were reported as the preferred modes of delivery, accompanied by supporting resources for professionals. However, the specific training needs of respondents varied. Specific topics for training that were commonly reported included body image and body esteem, extreme dieting, how to communicate with students about their eating behaviours, media literacy and social media, and foundational information about eating disorders. Practical strategies and resources were also identified as important education and training needs for school professionals in supporting eating disorder prevention. However, the specific details of the resources and strategies that would be most helpful were not clearly detailed in the responses, for example, “Training on the available resources and programs, and peer-reviewed research showing the efficacy of the resources/programs. Practical tools that have been shown to work.” R58 (Primary).

## 4. Discussion

This study aimed to determine the perceptions, knowledge, confidence, and needs of Australian school professionals regarding eating disorder prevention, which is currently underrepresented in the published literature, specifically within regional contexts. Most participants in this study felt they had a good knowledge of eating disorders and related risk factors and high willingness to implement prevention approaches. Respondents generally agreed that eating disorder prevention was part of their role, but many were not confident in their ability to deliver eating disorder prevention. This may indicate a potential gap between self-perceived knowledge of eating disorders and confidence in the actual implementation of such prevention interventions. It was also found that most respondents believed that the implementation of eating disorder prevention in schools was necessary and supported the use of a whole-school approach. However, fewer respondents agreed that all teachers should all be involved in eating disorder prevention interventions in schools, with workload and knowledge being common barriers reported. Despite a willingness to be involved in the prevention of eating disorders, there seems to be some inconsistencies in attitudes regarding the role of teachers and school professionals in relation to eating disorder prevention.

School professionals are important role models for students in promoting positive body image, self-esteem, and healthy behaviours [35,36]. In the current study, participants acknowledged their willingness and responsibility as role models and in eating disorder prevention. However, there were some inconsistencies in the perceived roles of school professionals, which may be due to the wide range of professional roles recruited to this study. While role modelling is important for all school professionals, other roles may differ based on qualifications, interests, and interactions with students. There must be a clear distinction between the primary prevention of eating disorders and the treatment of eating disorders, the latter falling within the scope of trained professionals such a psychologists [25]. Future programs may consider the identification of champions with a particular interest or skills in eating disorder prevention, as opposed to all school professionals, for instance, school counsellors and physical education teachers, who are likely to have greater interactions and chances to stimulate eating-disorder-related discussion with students [25]. As outlined previously, existing programs addressing body image and eating disorder prevention have predominantly been implemented by external providers [21] and have shown variable efficacy in improving eating disorder risk factors. To develop more efficacious and sustainable interventions, further in-depth qualitative work to better define the perceived roles and needs of school professionals in eating disorder prevention is warranted.

Most school professionals in this study perceived a need for eating disorder prevention in schools, similar to previous research [37]. However, eating disorder prevention was perceived by many participants as a low-to-moderate priority in the school in which they were working, and few participants reported appropriate structures or policies in place at their school to support eating disorder prevention. This may indicate that while there is considerable individual interest and willingness to participate in eating disorder prevention, school professionals may perceive the need for higher-level change and further organisational recognition of eating disorder prevention as a priority in schools. This highlights the need to better understand the needs and perceptions of education organisations, alongside that of individual school professionals. Despite this, many participants recognised existing structures such as PDHPE curricula, wellbeing, and mental health programs as appropriate ways to incorporate prevention content, which is similar to recommendations of integrating programs into existing classes from respondents in a previous study [37]. While school settings are recognised as appropriate settings for prevention, this may also indicate that future prevention interventions should consider a staged approach, prioritising schools with an expressed interest in eating disorder prevention in the first instance.

Compared to secondary schools, fewer primary school professionals reported the need for prevention approaches in their schools, which may reflect the lesser recognition of disordered eating in younger children as opposed to adolescents. Barriers including workload, perceived knowledge, and current resources were commonly expressed by participants across all school settings, particularly by those in primary school settings. These findings are similar to other studies that identified the lack of training and availability of resources as common barriers faced by teachers in schools [25]. This is likely attributable to the increasing expectations and crowded curriculum in Australian schools, which is similar to a previous study in which teachers experienced insufficient time for delivering activities in an eating disorder prevention program [38]. Taken together, this emphasises the importance of training and education to increase recognition of the importance of eating disorder prevention, particularly within settings capturing younger children, to promote prevention activities in younger ages before behaviours become entrenched in adolescence.

Participants reported the optimal delivery of prevention programs via a multimodal approach including the training of school professionals, complemented by delivery of materials by external providers. While few participants had participated in training relating to eating disorders, importantly, school professionals in this study who had received education or training in eating disorders reported being more confident in eating disorder prevention and their knowledge of eating disorders. Establishing partnerships with experts could assist with program development, implementation, and evaluation to progress real-world implementation as well as the eating disorder prevention research field [39]. Facilitators that are closer to student ages and circumstances have been reported to be better received [23], which was also expressed in the qualitative responses of the current study. Therefore, the provision of education by external providers could provide an opportunity to recruit facilitators that are likely to have greater uptake by students. Although it is feasible for teachers to deliver such eating disorder prevention programs in schools, a smaller effect has been observed in one study when an eating disorder prevention program was delivered by trained non-professionals [39]. Therefore, delivering prevention program with collaborative partnerships between school professionals and eating disorder experts could enhance program outcomes. As face-to-face and online training was found to be equally preferred by participants in this study, a mixed approach of face-to-face and online delivery for training for the delivery of eating disorder programs in schools should be considered to allow higher flexibility and accessibility of the program over a broad geographical location across various regional locations. Further in-depth information about the types of resources and training, guidelines, practical strategies, and specific knowledge to be delivered within eating disorder training and prevention programs is needed in future qualitative research.

This work has implications for the development of eating disorder intervention programs, as well as the design and dissemination of professional learning programs for school professionals. This study has revealed that teachers are interested in learning more about this topic, and that there is a demand for eating disorder prevention programs. In designing these programs, experts need to ensure that they create developmentally appropriate materials that teachers can deliver even if they do not have high levels of knowledge and confidence in teaching this topic, which can be achieved by creating resources such as videos and interactive web tools rather than relying on teacher-delivered information and discussion. Programs should also take a health-promoting schools approach in order to increase the number or schools that have policies in place around body image, weight, and eating disorders [40,41]. In designing professional learning programs, it is important that the role of school professionals in eating disorder prevention and the acknowledgement of the personal attitudes and behaviours of school professionals are considered [42].

This study is limited by the small sample size, which may be due to the competing priorities for school professionals in 2020 due to COVID-19, and may reflect the challenges of engaging school professionals in a survey of this nature, recruitment issues which are also reflected in similar studies [7]. However, the median age of participants was similar to the workforce development profile of the teaching profession in NSW, though there was a lower proportion of males and more participants with postgraduate qualifications [43]. The engagement of males in future research is important given the increasing prevalence of disordered eating behaviours in males. The study may also be limited by the self-reported nature of some questions, for example, self-perceived knowledge. It is also important to consider the fact that the teachers who responded to a survey about eating disorders might have an increased personal interest in the topic for a range of reasons [25]. Previous research has shown that teachers who are interested in this topic do tend to have a lived experience of body image problems and eating disorders [44]. As such, the sample of teachers in this survey, while important in gaining information about needs and preferences, may not represent the broader range of attitudes of teachers and school professionals in regional Australia. However, this study did successfully recruit school professionals from a range of years of experience, settings, and roles from classroom teacher to executive, which assists in providing pilot data from a range of perspectives for future research, and suggests that there is a need for and interest in eating disorder prevention that transcends school settings and roles.

## Figures and Tables

**Table 1:** Participant characteristics of regional Australian school professionals who completed the online needs assessment survey presented as frequency (%) (*n* = 51).

Participant Characteristic	*n* (%)	
Gender		
Male	7	(13.7%)
Female	44	(86.3%)
Age *Median (IQR)*	41 Range	(32–50) 23–71
Years of Experience *Median (IQR)*	9 Range	(4–17) 0.5–38
School Setting		
Primary	11	(21.6%)
Secondary	25	(49.0%)
Primary and secondary	15	(29.4%)
School Type		
Public/government	40	(78.4%)
Private	5	(9.8%)
Catholic	6	(11.8%)
Boarding	1	(2.0%)
Coeducational school	50	(98.0%)
Highest Level of Education		
School certificate	2	(3.9%)
Higher school certificate	0	(0%)
Diploma	4	(7.8%)
University	25	(49.0%)
Postgraduate qualifications	20	(39.2%)
School Role		
Classroom teacher	22	(43.1%)
Personal development, health, and physical education	6	(11.8%)
Head teacher	5	(9.8%)
Welfare of wellbeing	9	(17.6%)
Counsellor or psychologist	10	(19.6%)
Assistant or deputy	3	(5.8%)
Principal	2	(3.9%)
*Other*	4	(7.8%)

Data is presented as frequency (%) unless otherwise specified.

**Table 2:** Participant perceptions regarding eating disorder prevention in schools presented as frequency (%) (*n* = 51).

*n* (%)		
Self-Reported Knowledge of Eating Disorders		
Very poor	0	(0%)
Poor	3	(5.9%)
Average	17	(33.3%)
Good	29	(56.9%)
Excellent	2	(3.9%)
Self-Reported Confidence in Eating Disorder Prevention		
Not at all	2	(3.9%)
Not that confident	7	(13.7%)
Neutral	21	(41.2%)
Confident	20	(39.2%)
Extremely confident	1	(2.0%)
Willingness to Participate in Eating Disorder Prevention		
Not at all	0	(0%)
Not that	1	(2.0%)
Neutral	3	(5.9%)
Willing	19	(37.3%)
Extremely	28	(54.9%)
Perception that Prevention is Part of Your School Role		
Yes	41	(80.4%)
No	3	(5.9%)
Unsure	7	(13.7%)
Eating Disorder Prevention Programs Needed at School		
Yes	47	(92.2%)
No	4	(7.8%)
Current Programs for Eating Disorder Prevention at School		
Yes	12	(23.5%)
No	27	(52.9%)
Unsure	12	(23.5%)
Current Policies or Structures to Support Eating Disorder Prevention at School		
Yes	13	(25.5%)
No	9	(17.6%)
Unsure	29	(56.9%)

## Data Availability

Not applicable.
